# Regression of fetal vasculature and visual improvement in nonsurgical persistent hyperplastic primary vitreous: a case report

**DOI:** 10.1186/s12886-019-1173-3

**Published:** 2019-07-26

**Authors:** Jianqing Li, Jiaju Zhang, Peirong Lu

**Affiliations:** Department of Ophthalmology, the First Affiliated Hospital of Soochow University, 188 Shizi Street, Suzhou, 215006 People’s Republic of China

**Keywords:** Persistent hyperplastic primary vitreous, Persistent fetal vasculature, Congenital cataract, Tunica vasculosa lentis, Amblyopia

## Abstract

**Background:**

Persistent hyperplastic primary vitreous (PHPV) is a rare congenital developmental ocular disorder caused by incomplete regression of the embryonic hyaloid vasculature. Here we report a case of nonsurgical unilateral anterior PHPV that was managed by amblyopia treatment and resulted in an improvement of visual acuity and regression of the fetal vasculature.

**Case presentation:**

A three-year-old girl was diagnosed with unilateral anterior PHPV in the left eye, manifested with posterior pole cataract, posterior capsule opacification, tunica vasculosa lentis, and a floating hyaloid artery connected to the retrolental mass. The plaque was not large enough to fill the pupil, and conservative management along with amblyopia treatment was conducted. Nineteen months later, the visual acuity in the affected eye improved from 20/100 to 20/50 with correction, and the fetal vasculature regressed gradually and finally into a nonperfusion ghost vessel.

**Conclusions:**

In PHPV-affected children, regression of the fetal vasculature may be observed, and conservative management and amblyopia treatment may be helpful for visual improvement.

**Electronic supplementary material:**

The online version of this article (10.1186/s12886-019-1173-3) contains supplementary material, which is available to authorized users.

## Background

Persistent hyperplastic primary vitreous (PHPV), also known as persistent fetal vasculature, is an ocular developmental malformation characterized by the failure of the embryonic hyaloid vasculature to regress completely [1, 2]. The cases are typically sporadic and unilateral and can be further classified on the basis of location into anterior, posterior, and combined types [2, 3]. Anterior PHPV is the most common type, occurring in the anterior segment and involving a retrolental mass and cataract, while posterior PHPV is much less common. It is characterized as exhibiting one or more of the following features associated with an elevated vitreous membrane or stalk from the optic nerve: a retinal fold or retinal dysplasia, retinal detachment, or optic nerve hypoplasia [4].

The management of PHPV may involve either surgery or observation. Surgery is the mainstay of therapy in complicated cases [5], aiming to improve visual acuity and prevent the onset of ocular complications such as glaucoma, hemorrhage, and enucleation. However, intraoperative bleeding, postoperative hyphema, corneal decompensation, glaucoma, vitreous hemorrhage, and retinal detachment are common surgical complications [5–8]. Thus, despite the fact that many studies provide support for surgical treatment for PHPV, it may not be the optimal option for every patient. Goldberg [2] indicated in the 55th Jackson Memorial Lecture that many minimally affected eyes did not develop secondary complications and remained stable without surgical treatment, arguing in support of observation management for certain conditions.

In this case, we performed observation and amblyopia therapy on a three-year-old girl who suffered from unilateral anterior PHPV. During the 19 months’ follow-up, the visual acuity in her impaired eye improved from 20/100 to 20/50 with correction. The regression of the fetal vasculature was also observed in this unoperated PHPV case, which to the best of our knowledge had not previously been reported.

## Case presentation

A three-year-old Chinese girl came to our department in December 2016. She was born in a township hospital and had never received any physical examination until kindergarten admission. She was found to have amblyopia in the left eye at that time and was then diagnosed with congenital cataract at other hospitals, yet had never received any relevant intervention in the past. The girl was a full-term birth by a 36-year-old mother who was diagnosed with thyroid cancer 5 months after delivery. There was no history of similar complaints in the child’s family.

**Fig. 1:**
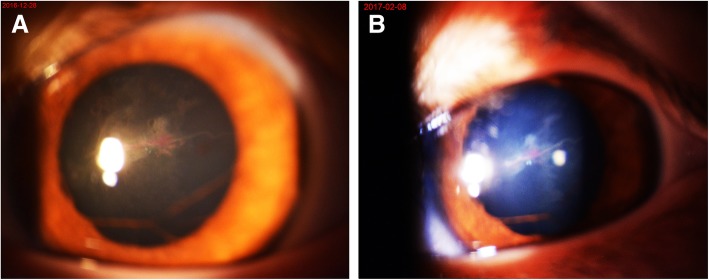
Anterior segment photography of the patient’s left eye. **a**. The photo taken at the first visit revealed a floating horizontal vessel that connected to the tunica vasculosa lentis. **b**. The image taken two months later indicated that blood flow in the hyaloid artery had slightly decreased

**Fig. 2:**
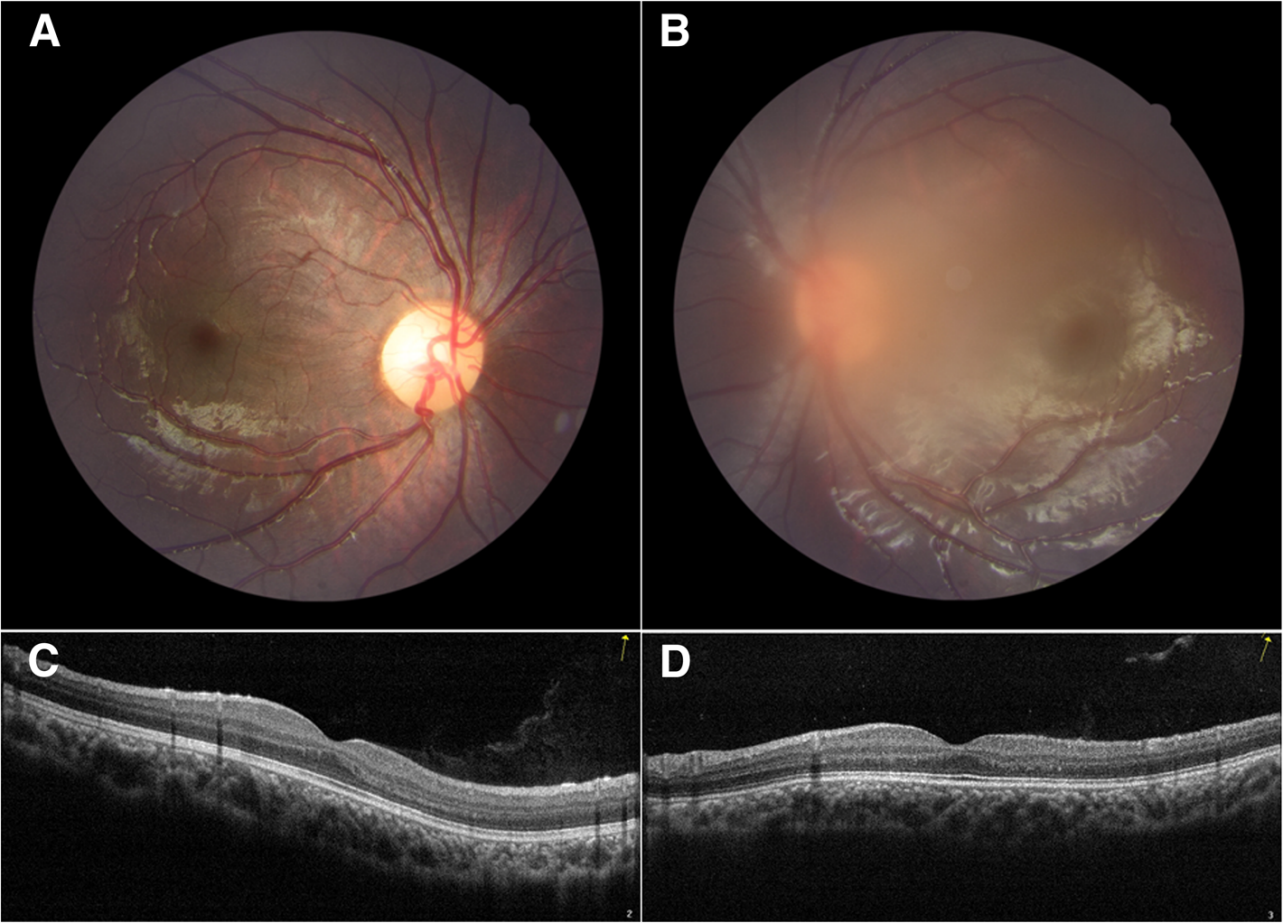
Fundus examination by fundus photography and optical coherence tomography taken at the first visit indicated no significant anatomical abnormalities. **a**. Fundus photography on the right eye. **b**. Fundus photography on the left eye. **c**. Optical coherence tomography on the right eye. **d**. Optical coherence tomography on the left eye

The girl’s physical and mental development was within normal range. Her best corrected visual acuity was 20/50 in the right eye and 20/100 in the left eye. The right eye examination was unremarkable, while in the left eye, slit lamp examination revealed posterior pole cataract and posterior capsule opacification as well as a floating horizontal vessel that connected to the tunica vasculosa lentis (Fig. 1a). Other ocular examinations did not identify any abnormalities; these examinations revealed normal intraocular pressure, cornea diameters and axial lengths, no dragged ciliary processes by ultrasound biomicroscopy, no fibrovascular stalk in the vitreous body by B-scan ultrasonography, and a healthy retina through ophthalmoscopy after mydriasis. Further fundus examination conducted by fundus photography and optical coherence tomography indicated no significant anatomical abnormalities (Fig. 2). Altogether, a diagnosis of unilateral anterior PHPV was made.

Considering that the fetal vasculature was attached to the retrolenticular fibrous membrane and the plaque was not large enough to fill the pupil [9], we decided to manage this case in a conservative manner instead of performing cataract surgery. The amblyopia was treated by 6 h of daily patching the fellow eye according to Amblyopia Preferred Practice Pattern of American Academy of Pediatric Ophthalmology [10]. Patching was conducted by applying an opaque patch to the contralateral eye and was closely monitored by her parents at home. Bimonthly follow-up examinations were arranged for her to monitor the visual acuity in both eyes as well as other ocular manifestations of the affected eye in order to adjust the treatment regimen timely.

**Fig. 3:**
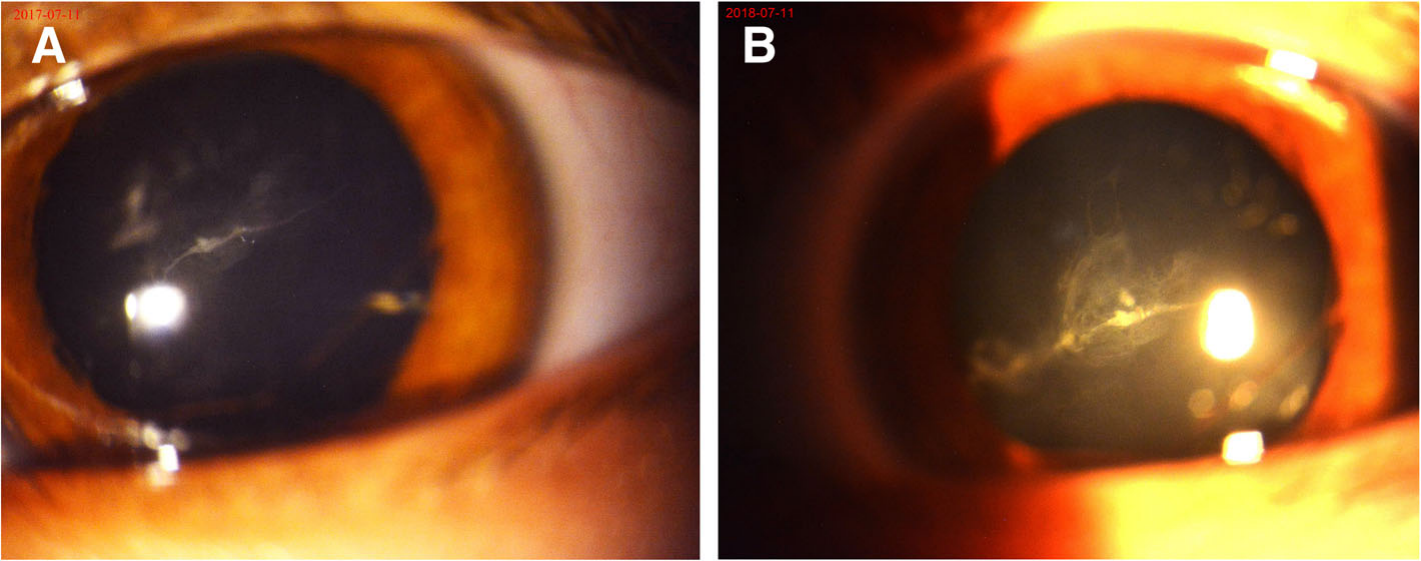
Anterior segment photography of the patient’s left eye. **a**. The picture taken at seven months’ follow-up revealed the nearly nonperfusion hyaloid artery. **b**. The photo taken at 19 months’ follow-up showed the regressed fetal vasculature as a nonperfusion ghost vessel


Two months later, no changes were noted in the child’s left eye except that the blood flow in the hyaloid artery had slightly decreased (Fig. 1b); thus, it was decided that the treatment was to be continued. On seven months’ follow-up, the displaced hyaloid artery was noted to be nearly a nonperfusion vessel (Fig. 3a). An additional movie file showed this artery in more detail (see Additional file 1).

**Additional file 1:** An additional movie file shows the regressed nonperfusion hyaloid artery in more detail. (MP4 10183 kb)


At 19 months’ follow-up, the improvement of the visual acuity of the child’s left eye to 20/50 with correction (+ 1.00D diopter cylindrical component at 85°) proved our treatment to be useful. The regression of the floating fetal vasculature into a nonperfusion ghost vessel was also observed (Fig. 3b). Comprehensive ocular examinations were conducted, and no ocular complications were detected.

Considering the patient’s age and visual acuity, she was advised to continue the treatment for amblyopia and the daily patching time was prescribed to be 2 h. Bimonthly follow-up was suggested, and the cataract would be treated with surgery if necessary.

## Discussion and conclusions

We reported a sporadic case of a three-year-old girl with unilateral anterior PHPV in the left eye, which was associated with improvement of visual acuity and the regression of the fetal vasculature after being managed conservatively by amblyopia therapy.

PHPV is managed either by surgery or by conservation. Several studies have compared ocular complications between these two managements. Alexandrakis [11], together with Federman and coworkers [12], reported a relatively greater incidence of complications in the nonsurgical group as compared to the surgical group, while Gulati et al. [9], Anteby and colleagues [13], and Scott and associates [14, 15] found that surgically treated PHPV eyes had more ocular complications, such as retinal detachment, glaucoma, and enucleation, than unoperated ones. Besides, the Infant Aphakia Treatment Study revealed that PHPV patients suffered from the relatively higher likelihood of postoperative adverse events than those without PHPV [16] Regarding visual improvement, Tartarella et al. [17] reported visual improvement in 80% of surgically treated patients, while unoperated patients did not show visual improvement, and approximately 40% exhibited visual deterioration. However, those nonsurgical patients did not receive amblyopia treatment. Roussat and colleagues [18] found that if the cataract was mild in the anterior form of PHPV, amblyopia treatment could be sufficient; however, if the cataract was dense, a lensectomy must be performed. What’s more, the visual acuity at the final visit varied largely from light perception to mild vision impairment (≥ 20/63 according to the World Health Organization classification method) in both surgical and nonsurgical treatment groups [9, 11, 19–24]. Therefore, the optimal management for PHPV depends on the individual case.

In our case, we decided to conduct conservative management along with amblyopia therapy, considering the following factors. Firstly, the left eye was minimally affected, since it suffered merely from anterior PHPV with congenital cataract and tunica vasculosa lentis, while surgery tended to be urgent in posterior types. The plaque was not large enough to fill the pupil, and the fundus was visible; thus amblyopia training had the potential for visual improvement. Besides this, the perfused hyaloid artery, which was attached to the posterior capsule, might easily bleed during the operation. Therefore, the girl was only treated for amblyopia by intermittent patching of the contralateral eye. Since some ocular complications such as secondary glaucoma [25] and intravitreal hemorrhage [26] might occur with conservative management, close follow-up needed to be conducted. Nineteen months later, the visual acuity in the left eye had improved from 20/100 to 20/50 with correction, and no ocular complications were observed. The fetal vasculature also regressed gradually and finally into a nonperfusion ghost vessel.

The intravitreal hyaloid vessels arise partly from the tunica vasculosa lentis and attach to the avascular retina between the ora and equator [27]. They reach a peak at 10 weeks’ gestation and undergo apoptosis at 5–6 months’ gestation, completing atrophy by the eighth month [28]. The failure of these vessels to regress completely leads to the formation of PHPV. After literature searching in English and Chinese on Pubmed, Embase, the Cochrane Libraty and Google, we believe that this article is the first clinical description of postnatal spontaneous regression of the fetal vasculature to the best of our knowledge. So far, there have been no publications on the mechanism of the postnatal spontaneous regression of the hyaloid vascular system in human because the hyaloid vessels tend to regress in utero, yet the involution occurs in the early postnatal period in mice [29]. The study groups of Lang RA [30], Lobov IB [31], Zhang H [32] and Kishimoto A [33] have studied this mechanism in neonatal mice and they have found that macrophages may play a central role in the regression of hyaloid vasculature involving the blocking of blood flow, the induction of apoptosis and the clearance of atrophic vessels. Besides, the Arf tumor suppressor gene [34], Norrie gene product [35] and some proapoptotic factors Bax, Bak [36] and Bim [37] have been discovered to be involved with postnatal fetal vasculature regression. What is more, neurons [38] as well as a progressive decrease in blood velocity in the hyaloid vessels [39] have been considered to be the triggering factors of the postnatal vessel regression. In this case report, we could not rule out the possibility that Arf tumor suppressor gene might play a role in the delay of fetal vasculature regression because although the child’s mother was diagnosed with thyroid cancer 5 months after delivery, she was likely to suffer from the cancer during her pregnancy which might influence the Arf tumor suppressor gene of the child. In addition, the other above mentioned mechanisms might also be of significance in the regression of hyaloid vasculature in the present case.

However, the main limitation in our case was that anesthesia was not performed on the child during examination despite of the child’s noncooperation. Therefore, the process was a bit difficult and the photos and video were not of the best quality. Nevertheless, the parents did not agree on the anesthesia thus we had to give up.

In summary, the management of PHPV should be decided according to individual cases. Our case does not support immediate surgery on minimally affected PHPV children; instead, conservative management and amblyopia treatment may be helpful for visual improvement or even fetal vasculature regression.

## Data Availability

All data supporting our findings are provided in the manuscript.

## Abbreviation

PHPV: Persistent hyperplastic primary vitreous
